# A young girl with an unusual cause of acute kidney injury: Questions

**DOI:** 10.1007/s00467-015-3170-y

**Published:** 2015-08-20

**Authors:** Mathijs Binkhorst, Kioa L. Wijnsma, Eric J. Steenbergen, Nicole C. A. J. van de Kar, Michiel F. Schreuder

**Affiliations:** 1 Department of Paediatrics, Radboud University Medical Centre, Amalia Children’s Hospital, Nijmegen, Netherlands; 2 Department of Paediatric Nephrology, Radboud University Medical Centre, Amalia Children’s Hospital, Nijmegen, Netherlands; 3 Department of Pathology, Radboud University Medical Centre, Nijmegen, Netherlands

**Keywords:** Acute interstitial nephritis, Hemorrhagic, Acute kidney injury, Child

## Case presentation

A previously healthy 3-year-old girl presented to the emergency department of a general hospital with high-grade fever, nausea, vomiting, diarrhea, and coughing. A week prior to presentation, she had low-grade fever, a sore throat, a red tongue, and bilateral purulent conjunctivitis. In between these two episodes, there was a relatively symptom-free interval. There were no explicit environmental exposures, such as contact with rodents. She did not use any medication, especially no antibiotics or non-steroidal anti-inflammatory drugs (NSAIDs).

At the emergency department, she showed signs of shock with a decreased level of consciousness, confusion, tachypnea (44–48 breaths per minute), tachycardia (151–162 beats per minute), and low blood pressure (62/46 mmHg). On physical examination, she displayed generalized exanthema with desquamation of the skin and lips, a red tongue, enlarged (almost kissing) tonsils, conjunctivitis, and cervical lymphadenopathy. Auscultation of heart and lungs was unremarkable. Abdominal examination revealed bilateral flank pain.

Fluid boluses were administered to stabilize the shock. There was no need for inotropic or vasopressor support. Antipyretics (acetaminophen) were given to lower the fever and, after collection of blood cultures, broad-spectrum intravenous cephalosporin antibiotics (ceftriaxone) were started. This resulted in improvement of her mental state and hemodynamic parameters. The next day, despite extensive fluid therapy, she was still oliguric and developed generalized edema. Laboratory tests showed acute kidney injury (Table 1). Therefore, she was transferred to the paediatric nephrology department of our university medical centre.

**Table 1:** Course of clinical and biochemical parameters in our patient

Value (units)	At presentation	At referral	At discharge	One month after discharge
Diuresis (ml/kg/h)	<0.1	<0.1	4.3	ND^a^
Creatinine (μmol/l)	273	308	35	26
eGFR (Schwartz)^b^ (ml/min.1.73 m^2^)	14	12	110	147
Urea (mmol/l)	19	22.7	4.8	4.4
Albumin (g/l)	24.4	20	ND	ND
CRP (mg/l)	382	286	45	ND
Hemoglobin (mmol/l)	6.1	5.5	6.7	6.3
WBC (× 10^9^/l)	43.9	34.1	21.3	15.2
Thrombocytes (× 10^9^/l)	409	224	681	636
Urinalysis				
• Erythrocytes/μl	50–100	>50	5–10	0
• Leucocytes/μl	>200	25–50	>50	5–10
• Protein (g/l)	3.0	ND	0.33	<0.1

*CRP* C-reactive protein;* eGFR* estimated glomerular filtration rate;* ND* not determined;* WBC* white blood cells;

^a^Parents reported moderate polyuria and nocturia; ^b^ Calculated with a k value of 36.5

On admission, she was clinically stable and normotensive. In order to determine the extent and course of her infection and kidney injury, we performed several laboratory tests, the results of which are shown in Table 1. Further laboratory evaluation was carried out to unravel the cause of her kidney injury. The results of this evaluation will be elaborated upon in the Answers section (http://dx.doi.org/10.1007/s00467-015-3171-x). A renal ultrasound was done, which showed both kidneys to be enlarged with a normal corticomedullary differentiation, normal flow in the renal artery and vein, and no dilatation or signs of urolithiasis.

**Fig. 1:**
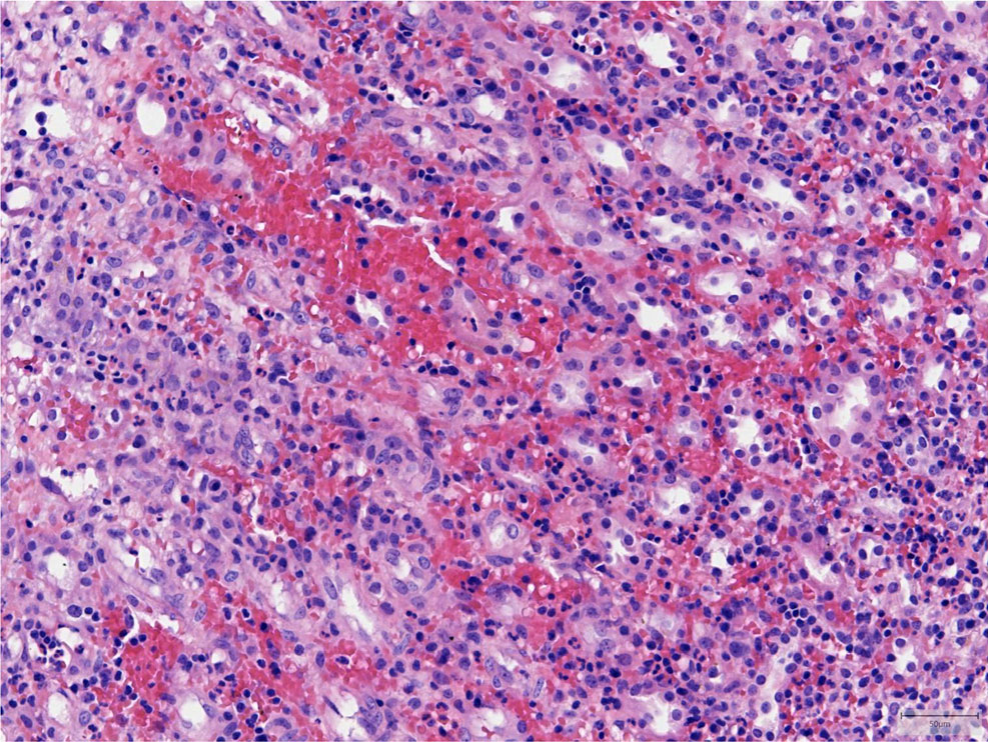
Renal biopsy specimen of our patient. Hematoxylin and eosin (H&E) staining, original magnification ×200. Medullary area with diffuse dense infiltrate with many neutrophils and a minority of mononuclear cells, including a few plasma cells. There is congestion of peritubular capillaries and extravasation of red cells, indicative of microvascular injury. Tubulitis is relatively mild and there are no viral inclusions or cytopathic changes

A rapidly progressive glomerulonephritis seemed likely considering the hematuria, proteinuria, edema, reduced renal function, and enlarged kidneys seen on ultrasound. A renal biopsy was performed and our patient was started on methylprednisolone pulse therapy for three consecutive days, followed by oral prednisone. The biopsy specimen was processed for light and immunofluorescence microscopy using standard techniques. Light microscopic sections showed on average 20 glomeruli, none obsolescent, all unremarkable. There were no abnormalities of the arteries and arterioles. A distinct interstitial infiltrate was seen, most prominent in the medullary areas of the biopsy, consisting of both mononuclear cells and neutrophils. Furthermore, the interstitium was markedly hemorrhagic with relatively mild tubulitis. A few intraluminal pus collections were noted. Viral inclusions and viral cytopathic changes were not detected. Immunofluorescence studies showed no staining for IgG, IgA, IgM, C1q, C3, or light chains. The biopsy was consistent with a diffuse hemorrhagic interstitial nephritis (Fig. 1).

The blood culture revealed a group A beta-hemolytic streptococcus (*Streptococcus pyogenes*), for which antibiotic therapy could be narrowed down to intravenous penicillin. Fever disappeared, skin and eyes improved, and C-reactive protein (CRP) and blood leukocytes declined. Following a regimen of fluid and dietary restrictions combined with methylprednisolone pulse therapy and subsequent oral prednisone, diuresis and overall clinical condition improved, renal function recovered, and hematuria and proteinuria subsided (Table 1). A short period of moderate polyuria occurred during recovery. After 11 days, our patient was discharged with a normal renal function, minimal residual proteinuria, and normal blood pressure. Follow-up has been uneventful so far.

## Questions

1. What is the differential diagnosis of acute interstitial nephritis (AIN)?
2. What is the differential diagnosis of hemorrhagic interstitial nephritis (HIN)?
3. How can streptococcal infection be related to acute kidney injury (AKI)?

